# Laparoscopic aspirator bracket: a new instrument facilitating the aspiration and exposure of operative field simultaneously in laparoscopic nephron-sparing surgery

**DOI:** 10.3389/fonc.2023.1216963

**Published:** 2023-08-16

**Authors:** Fengqi Yan, Xiaoliang Dou, Guangfeng Zhu, Qisheng Tang, Bo Zhang, Bo Zhao, Lei Yu, He Wang, Yong Wang

**Affiliations:** ^1^ Department of Urology, Tang Du Hospital, Air Force Military Medical University, Xi’an, Shaan’xi, China; ^2^ Department of Urology, Bao Ji People’s Hospital, Baoji, Shaan’xi, China; ^3^ Department of Urology, Xi Jing Hospital, Air Force Military Medical University, Xi’an, Shaan’xi, China

**Keywords:** laparoscopy, aspirator bracket, laparoscopic nephron-sparing surgery, operation time, warm ischemia time

## Abstract

**Background:**

This study aims to describe a novel laparoscopic aspirator bracket (LAB) and its use in laparoscopic nephron-sparing surgery (NSS) by a simple enucleation (SE) technique.

**Methods:**

A total of 123 renal tumor cases who underwent laparoscopic NSS via LAB or laparoscopic aspirator between July 2017 and April 2021 were retrospectively analyzed. General characteristics, perioperative data and postoperative follow-up data of patients were compared.

**Results:**

The application of LAB in laparoscopic renal tumor SE surgery shortened the operation time (88.58 ± 38.25 *vs*. 102.25 ± 35.84 min, *p* < 0.05) and improved the zero ischemia rate (18.75% *vs*. 3.39%, *p* < 0.05), shortened warm ischemia time (16.17 ± 5.16 *vs*. 19.39 ± 5.62 min, *p* < 0.05) and decreased intraoperative blood loss (166.19 ± 111.60 *vs*. 209.15 ± 127.10 ml, *p* < 0.05). In addition, the serum creatinine and eGFR values in the LAB group also showed faster and better renal function recovery.

**Conclusion:**

The new LAB could aspirate and expose the operative field with a single instrument. In operations that need to expose and aspirate simultaneously, such as in renal tumor simple enucleation, it could shorten operation time, reduce intraoperative blood loss and improve the postoperative renal function recovery.

## 1 Introduction

Over the past four decades, laparoscopic and robot-assisted laparoscopic operation have become the mainstay of urologic operations (1). However, the exposure of operative field during surgery is a constant problem, especially in surgical fields full of liquids, such as blood, urine, lymph, or melted fat. The surgeon has to manipulate a pair of laparoscopic forceps in one hand for exposure and a laparoscopic ultrasound knife or a pair of bipolar forceps in the other hand for hemostasis. To aspirate the liquids in the operative field, the surgeon needs to exchange the laparoscopic forceps for a laparoscopic aspirator, which will increase the operative time and bleeding volume. However, for some special operations, such as renal mass enucleation, a renal pelvic clamp is required to block the renal blood supply, and the elongated warm ischemia time (WIT) may result in worse renal function (2). Subsequently, the “zero ischemia” technique was introduced to eliminate renal ischemia caused by renal clamping. The term “zero ischemia” implies that tumor resection was successfully completed without hilar clamping and thus not subjecting the whole involved kidney to ischemic stress (3). However, the application of the zero ischemia technique also poses higher requirements for surgical technique, operating space, and surgical field of view. Some surgeons may add another trocar to allow the assistant surgeon to manipulate the aspirator, but the added surgical instruments may obstruct the operative space or even interfere with the surgeon’s operation, and the assistant trocar can add to the surgical incision (4).

How can the surgeon expose the operative field and aspirate the liquid simultaneously without congesting the operative field? Our idea was to combine the function of forceps and aspirator in one instrument. A new surgical instrument—laparoscopic aspirator bracket (LAB) made of silicone rubber—was designed. It was put at the end of the laparoscopic aspirator, thus making it applicable for surgical exposure and aspiration simultaneously, especially suitable for very narrow region and operative filed with full of liquids.

In this study, the new aspirator bracket was applied in a series of operations in combination with the laparoscopic aspirator, such as for simple laparoscopic renal tumor enucleation. Perioperative data were compared with traditional laparoscopic renal tumor enucleation.

## 2 Materials and methods

### 2.1 Clinical data

The study was approved by the Ethics Committee of Tang Du Hospital (April, 2017), and the written consent was signed by all the patients included in this study. All patients were evaluated according to institutional review board-approved protocols.

From July 2017 to April 2021, a total of 123 cT_1_-cT_2_ renal cell carcinoma patients were operated by laparoscopic surgery in 3 hospitals (Tang Du hospital, Xi Jing hospital and Bao Ji People’s Hospital) by 6 experienced urologists (Mr. Yong Wang, Mr. Bo Zhang, Mr. Jianjun Ma, Mr. Lei Yu, Mr. He Wang, and Mr. Bo Zhao). Each surgeon had more than 100 cases of experience in laparoscopic renal mass enucleation. Their peri-operative clinical data were documented and retrospectively analysed. All the patients were diagnosed with renal tumor mass by enhanced computed tomography (CT) scan before surgery. Sixty-four of them were operated with the LAB. Fifty-nine patients were operated with traditional laparoscopic aspirator (LA) and forceps.

The following demographic characteristics of the patients were collected: age, sex, body mass index (BMI), number of renal arteries, location of tumor, size of tumor, Radius Exophytic Nearness Anterior/Posterior Location (RENAL) score and tumour histology. In addition, the operation time, WIT, blood loss volume, intraoperative transfusion, complications, and pseudo capsule damage were recorded and analysed. Operation time was defined as the time from skin incision to closure, and WIT was defined as the time from clipping the renal artery to releasing the clip. All patients were followed up for at least 1 year after surgery, the serum creatine and eGFR values of patient were measured and analysed before surgery and at the 3rd, 6th and 12th months after surgery respectively.

### 2.2 Surgical instruments

**Figure 1:**
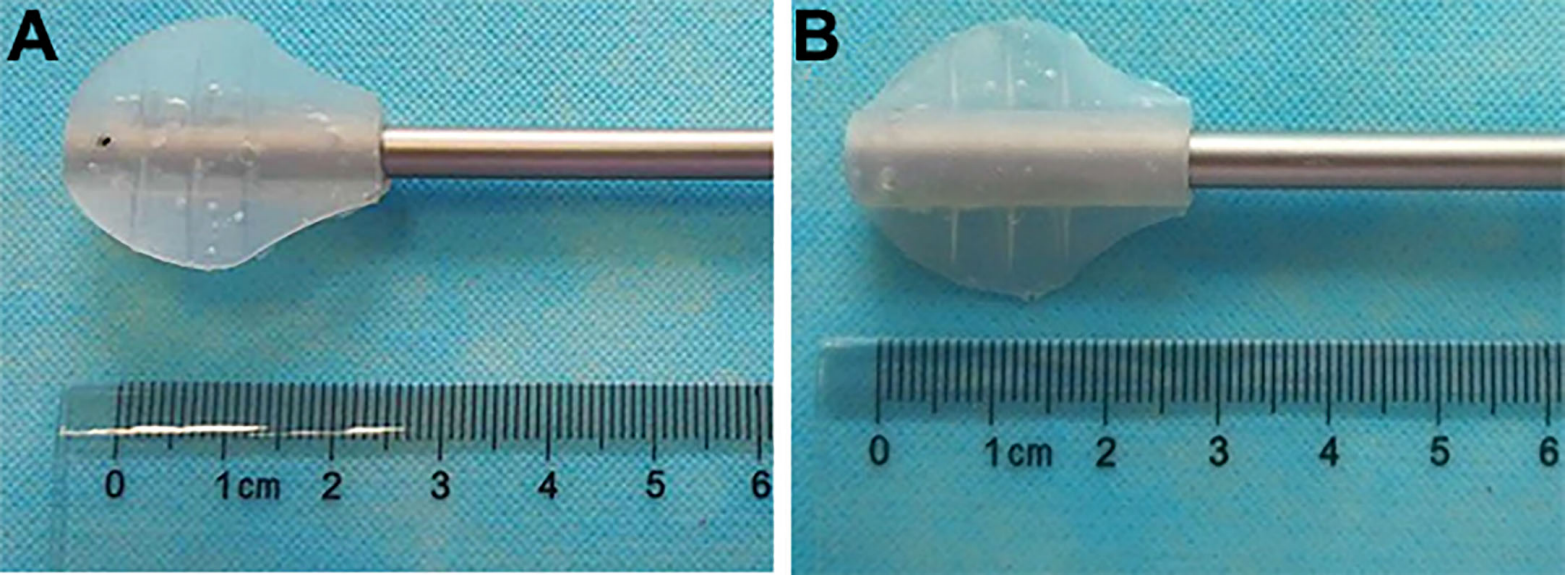
The laparoscopic aspirator bracket (LAB). **(A)**: The front side of the LAB; **(B)**: The back side of the LAB.

The LAB is an invention (ZL 2016 2 0736850.2 from State Intellectual Property Office of P.R. China) developed by Mr. Yong Wang (**Figures 1A, B**). It is made of S820 silica gel (Xi Rui Bo Technology Co lTD, Wu Han, P.R. China) by Chang Ping Industrial Incorporation (Xing Ping County, Shaan’xi Province, P.R. China). It needs a 12-mm standard laparoscopic trocar to pass through. In both groups, all the laparoscopic instruments were the same. The 5-mm laparoscopic aspirator (No. 101.149) was made by Kang Ji Medical Instruments Ltd. (Hang Zhou, Zhe Jiang Province, P.R. China).

### 2.3 Surgical procedure

The tumor was enucleated with a simple enucleation technique (2). That is, it was incised to open the parenchyma along the tumor margin, bluntly separated the tumor and renal parenchyma along the pseudocapsule by closed laparoscopic scissors. To expose the cleavage plane, the tumor was pushed aside with either the LAB (LAB group) or the forceps (traditional suction group; TS group). The large vessels traversing this surgical plane were cutted with laparoscopic scissors and then clipped in the surgical plane when needed. Then, the whole tumor was enucleated from the kidney along the cleavage plane. The bleeding site was ablated with bipolar forceps. Running suture was used for the tumor bed, with 2-0 V-Loc™ sutures (Medtronic, Inc., Shang Hai, P.R. China). For high RENAL score tumors (5), a laparoscopic ultrasound was used to locate the tumor and determine the distance between the tumor and renal vessels or the collection system. The peri-operative data were documented.

**Figure 2:**
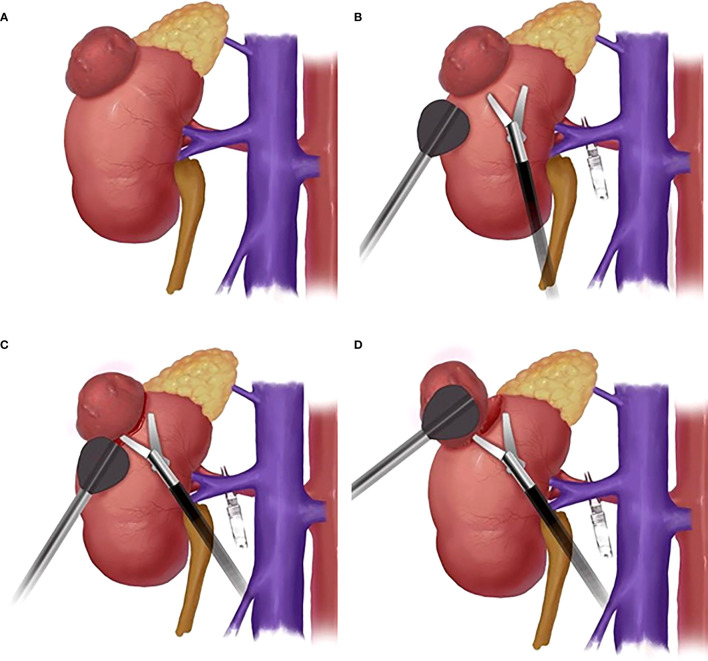
The schematic diagram of surgical procedure. **(A)**: Schematic diagram of kidney tumor; **(B)**: Schematic diagram of the position of LAB and laparoscopic scissors; **(C)** Schematic diagram of LAB-assisted scissors to cut open renal capsule; **(D)** Schematic diagram of LAB combined with scissors cutting and blunt separation of kidney tumors.

For the TS group, the surgeon (right-handed) had laparoscopic forceps in the left hand and the laparoscopic scissors in the right hand for cutting. When too much blood was seen in the surgical cleavage plane between tumor and parenchyma, the forceps were withdrawn and the aspirator was inserted (**Figure 2A**). If the surgical cleavage plane was clear, the aspirator was withdrawn and the scissors were inserted. The whole tumor was enucleated by a combination of cutting and blunt separation by the scissors (2).

For the LAB group, before enucleation of the renal tumor, the bracket was put on the end of the laparoscopic aspirator. The surgeon had the LAB in one hand for exposure and aspiration and the laparoscopic scissors in the other hand for cutting (**Figure 2B**). The renal capsule was cut open with the scissors (**Figure 2C**) and the tumor was pushed aside with the LAB. The cleavage plane was found by a combination of cutting and blunt separation with the scissors (**Figure 2D**), and the blood was aspirated with the aspirator whenever needed. The whole tumor was then enucleated along the cleavage plane (Supplementary Video: https://drive.google.com/file/d/1WYmkkPLgMqcjxcgRFs_CgBajwLLlxBlw/view?usp=sharing).

### 2.4 Statistical analysis

Descriptive analyses of patient characteristics, perioperative data, and postoperative follow-up data were conducted, including central tendency and dispersion (mean ± standard deviation [SD]), or median and frequency distribution. The outcomes were analysed using a t-test or χ^2^ test, and a *p*-value < 0.05 was considered statistically significant. SPSS version 18.0 (SPSS, Inc., Chicago, IL) was used for statistical analysis.

## 3 Results

The demographic characteristics of the patients are shown in **Table 1**. A total of 123 patients were enrolled in this study. The patients were 58.89 ± 10.55 years old, with a median age of 60 years. Among all patients, 87 were males, and 36 were females. The BMI of patients was 24.69 ± 4.18 kg/m^2^. Of all the patients, 61 patients had the tumor on the right side and 62 on the left side. In addition, 13 of the 123 patients had two renal arteries supplying blood to the tumor. The tumor average diameter_max_ is 4.15 ± 1.40 cm, and the RENAL score was 6.19± 1.64 points. The pathological results showed that among 123 patients, ccRCC accounted for 104, Papillary type accounted for 11, Chromophobe accounted for 6, and Oncocytoma accounted for 2. Patients were randomly assigned to the LAB group and the TS group, of which 64 were in the LAB group and 59 were in the TS group. There were no difference in demographic characteristics between the two groups of patients (**Table 1**).

**Table 1:** Baseline characteristics for patients involved in this research.

Item	All (*N* = 123)	LAB group (*n* = 64)	TS group (*n* = 59)	*p* value
Age (years)				
Mean ± SD	58.89 ± 10.55	58.89 ± 10.42	58.86 ± 10.77	0.989
Median	60.0	60.5	59.0	
Sex				
Male	87	44	43	0.615
Female	36	20	16	
**BMI (Kg/m^2^)**	24.69 ± 4.18	24.38 ± 4.60	25.02 ± 3.68	0.400
**Number of** **renal arteries**	1:(110/123) 2:(13/123)	1:(56/64) 2:(8/64)	1:(54/59) 2:(5/59)	0.4682
Side				
Right	61	33	28	0.649
Left	62	31	31	
**Mean Diameter_max_ (cm)**	4.15 ± 1.40	4.17 ± 1.58	4.12 ± 1.17	0.864
**RENAL score**	6.19± 1.64	6.17 ± 1.62	6.20 ± 1.67	0.916
R	1:(64/123) 2:(51/123) 3:(8/123)	1:(33/64) 2:(26/64) 3:(5/64)	1:(31/59) 2:(25/59) 3:(3/59)	0.827
E	1:(76/123) 2:(36/123) 3:(11/123)	1:(39/64) 2:(20/64) 3:(5/64)	1:(37/59) 2:(16/59) 3:(6/59)	0.825
N	1:(74/123) 2:(27/123) 3:(22/123)	1:(37/64) 2:(16/64) 3:(11/64)	1:(37/59) 2:(11/59) 3:(11/59)	0.670
A	A:(30/123) P:(23/123) Other(70/123)	A:(16/64) P:(11/64) Other(37/64)	A:(14/59) P:(12/59) Other(33/64)	0.904
L	1:(55/123) 2:(53/123) 3:(5/123)	1:(32/64) 2:(29/64) 3:(3/64)	1:(23/59) 2:(24/59) 3:(2/59)	0.925
Tumour histology				
ccRCC	104	56	48	0.760
Papillary type	11	5	6	
Chromophobe	6	2	4	
Oncocytoma	2	1	1	

LAB, laparoscopic aspirator bracket; TS, traditional suction; R, size criteria; E, endophytic/exophytic; N, nearness to sinus or calyx; A, anterior or posterior; L, location relative to polar line; The outcomes of frequency distribution were analyzed using χ^2^ test and the dispersion data (mean ± standard deviation [SD]) was analyzed using t-test. p-value refers to the comparison between LAB group and TS group, and p < 0.05 was considered statistically significant.

**Figure 3:**
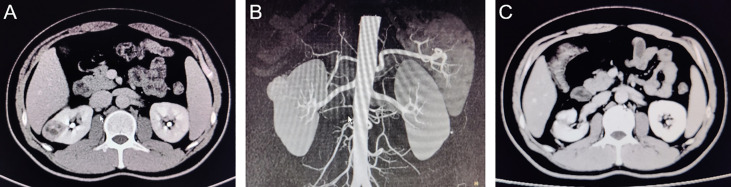
CT images of typical cases of partial nephrectomy with zero ischemia in LAB group. **(A)**: Preoperative coronal CT image; **(B)**:Pre-operative sagittal CT image; **(C)**: 3 months postoperative CT image.

All the procedures were performed successfully in 3 hospitals by 6 experienced urologists, and the operative data of the patients from two groups were compared. As shown in **Table 2**, there were no differences in intra-operative transfusion rate, perioperative complications and pseudo capsule damage between two groups. However, the operative time was shorter in the LAB group (88.58 ± 38.25 min) compared to the TS group (102.25 ± 35.84 min). Moreover, only 2 of the 59 patients (3.39%) in the TS group achieved zero ischemia, while 12 of the 64 patients (18.75%) in the LAB group achieved zero ischemia (**Table 2**, **Figure 3**). The WIT (Excluding zero ischemia cases) of the TS group was 19.39 ± 5.62 min, while the WIT of the LAB group was only 16.17 ± 5.16 min, which was also shorter than that of the TS group (*p* < 0.05). In addition, the blood loss in LAB group was 166.19 ± 111.60 ml, which was significantly lower than that in the TS group (209.15 ± 127.10 ml, *p* < 0.05).

**Table 2:** Results for the laparoscopic renal tumour enucleation.

Item	LAB group (*n* = 64)	TS group (*n* = 59)	*p* value
Operation time (min)	88.58 ± 38.25	102.25 ± 35.84	0.043
Number of Zero ischemia (%)	12 (18.75%)	2 (3.39%)	0.007
Warm ischemia time (min) (Excluding zero ischemia cases)	16.17 ± 5.16 (*n* = 52)	19.39 ± 5.62 (*n* = 57)	0.002
Blood loss (ml)	166.19 ± 111.60	209.15 ± 127.10	0.048
Intraoperative transfusion (%)	3 (4.69%)	2 (3.39%)	0.716
Complication (%)	0 (0%)	1 (1.69%)	0.296
Pseudo capsule damage (%)	0 (0%)	1 (1.69%)	0.296

In order to further compared the recovery of renal function of patients between two groups, the values of serum creatinine and eGFR were measured and analyzed. As shown in **Table 3**, there were no difference in serum creatinine and eGFR values of patients in the TS group and the LAB group before surgery. However, with a regular follow-up of 12 months, the serum creatinine value of the patients in the LAB group had a quicker recovery at 3rd and 6th month after the surgery when compared with the TS groups (3rd month: 97.17 ± 19.61 *vs.* 103.67 ± 16.42 mg/dl, *p* < 0.05; 6th month: 95.13 ± 19.07 *vs.* 101.46 ± 15.50 mg/dl, *p* < 0.05). In addition, the eGFR value of patients in TS group decreased from 96.05 ± 12.48 ml/min/1.73m^2^ before surgery to 87.23 ± 12.46 ml/min/1.73m^2^ at the 3rd month after surgery, while that in the LAB group decreased from 97.39 ± 11.49 to 93.43 ± 11.53 ml/min/1.73m^2^ only (**Table 3**). Moreover, compared with the TS group, the LAB group also showed quicker recovery of eGFR value at 6 and 12 months after surgery (95.40 ± 11.52 *vs.* 88.96 ± 12.06 ml/min/1.73m^2^, *p* < 0.05; 97.01 ± 11.34 *vs.* 91.70 ± 12.22 ml/min/1.73m^2^ *p* < 0.05; **Table 3**).

**Table 3:** Perioperative renal function of different groups.

Item	LAB group (*n* = 64)	TS group (*n* = 59)	*p* value
Serum creatinine(mg/dl)			
Preoperative	93.39 ± 19.90	94.15 ± 15.86	0.816
3 month	97.17 ± 19.61	103.67 ± 16.42	0.049
6 month	95.13 ± 19.07	101.46 ± 15.50	0.047
12 month	93.53 ± 18.94	98.61 ± 16.11	0.113
eGFR (ml/min/1.73m^2^)			
Preoperative	97.39 ± 11.49	96.05 ± 12.48	0.537
3 month	93.43 ± 11.53	87.23 ± 12.46	0.005
6 month	95.40 ± 11.52	88.96 ± 12.06	0.003
12 month	97.01 ± 11.34	91.70 ± 12.22	0.014

## 4 Discussion

Surgical exposure in a narrow operative space is a constant problem in laparoscopic operations. Surgeons have developed many new surgical methods and instruments to improve it, such as the magnetic anchoring guidance system (MAGS) (6), suture suspension (7), intra-abdominal exposure instrument (8), and the natural orifice approach (9), but each method has its pros and cons.

MAGS is a promising complex technique. Levita Magnetics’ Surgical System got approval for laparoscopic gallbladder removal from the Food and Drug Administration of the United States in 2016. It could perform the intraperitoneal operation through a single incision (around 30 mm). However, to put in the magnetic anchored laparoscope, the surgeon needs a 20–35 mm trocar, which harms the cosmetic effect of the laparoscopy and increases the incision-related incident ratio. In addition, it needs special magnetic anchored laparoscopic instruments, which greatly limits its clinical application (10). Suture suspension or fixation is a convenient method for laparoscopy surgeons that does not require additional special instruments. This technique allows laparoscopic surgeons to suspend any point without adding a trocar or any obvious scar and thus decreases the difficulty of laparoscopic ureteropelvic anastomotic suture (11), but it does not help clear the liquids in the operative field. The intra-abdominal exposure instrument was a new invention designed by Mr. Qingyi Zhu (CN201620327288.8) (8). This hairpin-shaped intra-abdominal exposure instrument could help expose the operative field by pushing the tissue aside. It is useful in many operations, especially for very narrow and hard-to-expose places, such as single-incision, retroperitoneal, and laparoscopic adrenalectomy. However, it cannot aspirate the liquids in the operative field and, once fixed, is hard and time-consuming to adjust. To operate or retract a specimen through the natural orifice approach is also quite efficient. It uses the natural orifice, such as the urethra (9) or vagina (12), to help the surgeons perform the operation or to retract a specimen. However, it is limited to some special operations, such as radical prostatectomy, or for female patients only.

For some laparoscopic operations, such as partial nephrectomy or simple renal tumor enucleation, the operative space is rather limited and full of liquids, such as blood, urine, and lymph. In addition, operation time, especially the warm ischemia time, is very limited (13). In such situations, anatomic exposure and liquid aspiration are of equal importance (14). However, to aspirate liquid and expose the anatomic plane simultaneously, such as the plane between the pseudocapsule and the renal parenchyma, challenges hand skill. The surgeon has two options: (A) To hold the laparoscopic scissors or ultrasound knife in one hand, with the aspirator or forceps in the other hand in an alternative mode. The time to change and position instruments may lengthen the WIT. (B) The surgeon could hold the laparoscopic forceps in one hand for exposure and have an assistant surgeon manipulate the aspirator. In this kind of layout, the forceps, scissors, and aspirator may congest the anatomic plane, making it difficult to operate. The assistant also needs a learning curve to coordinate with the operative surgeon skillfully, which may affect the WIT (15). In addition, the fourth trocar may add to abdominal wall trauma.

To solve this problem, the LAB was invented. It combines the functions of forceps and aspirator, and compared with forceps and aspirator, LAB has the following four advantages. (A) It can aspirate liquid and expose the field with a single instrument, thus saving WIT and increasing the surgeon’s confidence for a zero ischemia operation. (B) It combines the functions of forceps and aspirator, thus reducing the use of ancillary operating instruments, which in turn saves operative space, and also reducing the use of additional assistant trocars.(C) Its design permits the surgeon to aspirate and expose the field by himself or herself without additional assistant surgeon, which facilitates the operation. (D) The LAB’s material (silicone rubber) helps protect the pseudocapsule of the tumor and lowering the chance of pseudocapsule damage. In simple laparoscopic tumor enucleation, the operator has to substitute forceps for aspirator constantly if just 3 trocars are used. During forceps withdrawal and aspirator insertion, bleeding in the surgical plane continues. As a result, surgeons have to repeat the aspirate-expose-cut-aspirate procedure, which prolongs the operative time and WIT and increases intraoperative blood loss. LAB combines the functions of forceps and aspirator, using it can greatly reduce the number of surgical instrument changes during surgery, thereby shortening the operation time and reducing intraoperative bleeding. Consistent with our assumption, the results of our retrospective analysis showed that the operative time and WIT in the LAB group were shorter than those in the TS group, and the blood loss also significantly reduced in the LAB group.

As we all know, in addition to the complete removal of the tumor, the most important principle of PN is to protect the renal function as much as possible. Therefore, the monitoring of postoperative renal function indicators is also a key indicator for evaluating the success of the operation. In recent years, the long-term implications of decreased renal function as a result of PN have been increasingly recognized, and various studies elucidate causes of decreased renal function after PN have been conducted. In addition to the loss of function associated with nephrectomy, at one time ischemia is considered by many researchers to be the most important factor affecting postoperative renal function (13). The article published in *European Urology* in 2010 by Prof. R. Houston Thompson proposed that every additional minute of warm ischaemia during PN for tumour in a solitary kidney correlated with a 5% increased risk of AKI, and a 6% increased risk of new-onset stage 4 chronic kidney disease (CKD) (16). The view that “every minute counts when the renal hilum is clamped during partial nephrectomy” has long been the dominant view in the academic community. However, in recent years, with the collection of long-term postoperative follow-up data on partial nephrectomy patients and the exclusion of other confounding factors, the significance of the effect of ischemia type and duration on long-term renal function has been questioned (17). For example, in an article published in *European Urology* by Prof. Alessandro Volpe concluded that “the WIT less than 25 minutes does not significantly affect long-term renal function “ (18). In conclusion, the effect of WIT on renal function after PN remains an important topic of debate. In this study, we found that although creatinine and other renal function parameters increased more rapidly in the TS group compared with the LAB group in the short term, they gradually converged between the two groups as the follow-up period was extended. This means that patients in the LAB group achieved better and faster recovery of renal function compared to those in the TS group, but overall there was no significant difference in long-term renal function between the two groups. We consider this may be related to the higher zero ischemia rate and shorter operative time and WIT in the LAB group.

In conclusion, our study shows that LAB can realize exposes and aspirates simultaneously by controlling a single instrument without occupying the surgical space. It is especially suitable for difficult operations that need to be performed in very narrow fields full of liquids such as blood, urine, and lymph. It has the potential to be used in robot-assisted surgery. Our results proved that the LAB can shorten operation time and WIT in renal tumor enucleation operations. In addition, it can be applied in many other operations, such as laparoscopic or robot-assisted laparoscopic radical prostatectomy and renal tumor embolus extraction.

## Data availability statement

The raw data supporting the conclusions of this article will be made available by the authors, without undue reservation.

## Author contributions

FY, XD, GZ and HW contributed to the conception and design of the study and developed the study protocol. YW, XD and GZ are responsible for the recruitment of subjects. QT, BZn, LY and BZo are responsible for the management of the trial, collection and analysis of the data. YW contributed to the administrative, technical, material support and obtaining funding. All authors contributed to modification of the original protocol and all authors read and approved the final manuscript.
